# Defining multiple, distinct, and shared spatiotemporal patterns of DNA replication and endoreduplication from 3D image analysis of developing maize (*Zea mays* L.) root tip nuclei

**DOI:** 10.1007/s11103-015-0364-4

**Published:** 2015-09-22

**Authors:** Hank W. Bass, Gregg G. Hoffman, Tae-Jin Lee, Emily E. Wear, Stacey R. Joseph, George C. Allen, Linda Hanley-Bowdoin, William F. Thompson

**Affiliations:** Department of Biological Science, Florida State University, 319 Stadium Drive, King Life Sciences Building, Tallahassee, FL 32306-4295 USA; Department of Plant and Microbial Biology, North Carolina State University, Raleigh, NC 27695-7612 USA; Department of Horticultural Science, North Carolina State University, Raleigh, NC 27695-7609 USA

**Keywords:** Cell cycle, DNA synthesis, Grass

## Abstract

Spatiotemporal patterns of DNA replication have been described for yeast and many types of cultured animal cells, frequently after cell cycle arrest to aid in synchronization. However, patterns of DNA replication in nuclei from plants or naturally developing organs remain largely uncharacterized. Here we report findings from 3D quantitative analysis of DNA replication and endoreduplication in nuclei from pulse-labeled developing maize root tips. In both early and middle S phase nuclei, flow-sorted on the basis of DNA content, replicative labeling was widely distributed across euchromatic regions of the nucleoplasm. We did not observe the perinuclear or perinucleolar replicative labeling patterns characteristic of middle S phase in mammals. Instead, the early versus middle S phase patterns in maize could be distinguished cytologically by correlating two quantitative, continuous variables, replicative labeling and DAPI staining. Early S nuclei exhibited widely distributed euchromatic labeling preferentially localized to regions with weak DAPI signals. Middle S nuclei also exhibited widely distributed euchromatic labeling, but the label was preferentially localized to regions with strong DAPI signals. Highly condensed heterochromatin, including knobs, replicated during late S phase as previously reported. Similar spatiotemporal replication patterns were observed for both mitotic and endocycling maize nuclei. These results revealed that maize euchromatin exists as an intermingled mixture of two components distinguished by their condensation state and replication timing. These different patterns might reflect a previously described genome organization pattern, with “gene islands” mostly replicating during early S phase followed by most of the intergenic repetitive regions replicating during middle S phase.

**Electronic supplementary material:**

The online version of this article (doi:10.1007/s11103-015-0364-4) contains supplementary material, which is available to authorized users.

## Introduction

Much of our knowledge of eukaryotic replication timing comes from analyses of yeast and animal cell culture systems, where complex temporal programs have been described (Jackson et al. 2012; Rhind and Gilbert 2013). Within S phase, different replicon populations are active at different times, and the temporal order of replication appears to follow a well-defined program. The replication time for a given region has been associated with transcriptional potential, epigenetic state, and subnuclear localization (Gilbert et al. 2010; Gindin et al. 2014; Pope and Gilbert 2013; Ryba et al. 2010). Resolving relationships between DNA replication, epigenetics, and chromatin structure is a major challenge in modern cell biology (Alabert and Groth 2012; Aparicio 2013; Julienne et al. 2013; Nordman and Orr-Weaver 2012; Pope and Gilbert 2013).

Studies of mammalian DNA replication have revealed changes in the spatial distribution of DNA synthesis through S phase (Kennedy et al. 2000; Nakayasu and Berezney 1989; O’Keefe et al. 1992; van Dierendonck et al. 1989). Although cytologically-defined substage patterns differ across studies, several features common to most mammalian replication patterns have emerged [types I–V, as summarized by Zink (2006)]. First, DNA synthesis during early S phase occurs at many foci widely distributed across the nucleoplasm. Second, DNA synthesis during middle S phase concentrates strikingly in perinuclear and perinucleolar regions. Finally, during late S phase, DNA synthesis is observed mainly at major heterochromatic sites. Replication factories, marked by PCNA, exhibit a similar progression of nuclear localization patterns (Leonhardt et al. 2000). Distributed replication in early S phase and an association of late replication with heterochromatin have also been reported in plants (Samaniego et al. 2002; Sparvoli et al. 1994), but the limited number of plant systems examined to date do not show the perinuclear and perinucleolar replication characteristic of mammalian middle S phase.

In stark contrast to the abundant reports for animal and fungal systems, few publications have addressed replication timing in plants. In the 1960s and 1970s, Van’t Hof and colleagues published reports that focused primarily on early versus late replication in various plant species (for example, Van’t Hof and Bjerknes 1979). More recently, Lee et al. (2010) used pulse labeling, flow cytometry, and tiling microarrays to identify over 150 putative replicons on Arabidopsis chromosome 4. That study’s comparative analysis of early, middle, and late replication revealed nearly identical patterns for early and middle S phase, suggesting that there are only two major replication phases in Arabidopsis. However, because Arabidopsis has an unusually small genome with relatively few repeat sequences, the relevance of these observations to more complex genomes like those of major crop plants is unknown. Samaniego et al. (2002) described patterns of BrdU incorporation into pulse-labeled onion cells, but reports using higher resolution, modern labeling techniques to examine more detailed spatiotemporal patterns are lacking, as are any reports for model systems such as maize. Furthermore, little is known about replication programs in endocycling cells, which occur normally in many developing plant tissues. Among the plant species that have been used for DNA replication studies, maize has a moderately large genome with a C-value of 2.7 pg (http://data.kew.org/cvalues/), comparable to that of the human genome (3.5 pg). In comparison, the Arabidopsis genome is particularly small (0.3 pg), whereas pea (*Pisum sativum* 4.9 pg) and onion (*Allium cepa* 17 pg) have slightly or considerably larger genomes. Carefully determining the temporal and spatial properties of DNA replication within the nucleus of a model system such as maize is critical to consolidate the structure–function relationships operative in plant genomes for which gene expression and epigenetic data are rapidly accumulating.

We recently developed a system [reviewed by Bass et al. (2014)] to analyze spatial and temporal aspects of DNA replication in maize (*Zea mays* L.) root tips pulse-labeled with the thymidine nucleoside analog, 5-ethynyl-2′-deoxyuridine (EdU). In contrast to animal DNA replication systems, which use cells in tissue culture, this system allows us to analyze DNA replication as it occurs in naturally developing, intact organ meristems. In addition, the moderately large, complex maize genome offers an excellent combination of cytology and genomics. The sequenced reference genome (inbred line B73) comprises ten metacentric chromosomes with genotype-specific heterochromatic knobs and a single rDNA locus on chromosome 6 (Birchler and Bass 2009; Schnable et al. 2009). We used high-resolution optical sectioning microscopy of *in planta* pulse-labeled nuclei to carry out 3D analysis of DNA replication in relation to bulk chromatin distribution in maize root tip cells. This approach uncovered spatial patterns of DNA replication that distinguish early from middle S phase in nuclei from mitotic cells, with the patterns persisting in nuclei from cells that entered the endocycle without passing through mitosis.

## Results

### Isolating nuclei in early, middle or late S phase of the mitotic cycle or endocycle

**Fig. 1:**
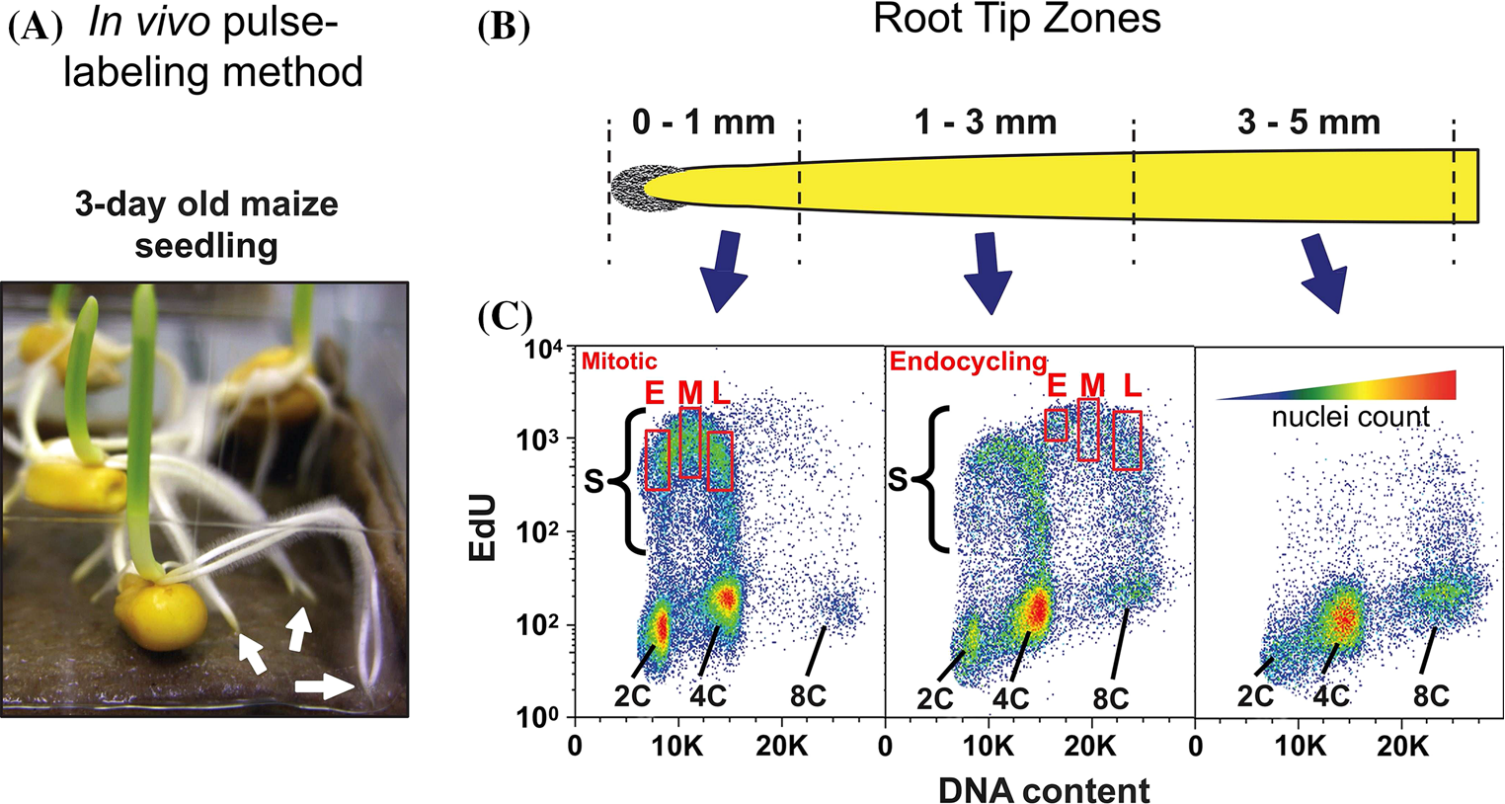
Pulse-labeling and flow cytometry gating used to collect early, middle, and late S-phase nuclei from mitotic and endocycling maize root tip cells. **a** Maize root tips from 3-day old seedlings were pulse-labeled with EdU for 20 min, followed by root tip harvest (*arrows*) and formaldehyde fixation and nuclei isolation. **b** Root tips were cut into sized segments (*top diagram*) for nuclei preparations. The root cap (*gray*, *left* end of zone 0–1 mm) covers the root apical meristem, from which cells divide and eventually differentiate (from *left* to *right*). EdU incorporated into DNA was conjugated to a fluorescent probe (A-488) using click chemistry, and nuclei were counter-stained with DAPI. **c** Nuclei were analyzed by flow cytometry using 355 nm (UV) and 488 nm (*blue*) lasers. Bivariate plots of DNA content (X-axis, DAPI fluorescence with emission filter 460 ± 50 nm) and EdU incorporation (Y-axis, Alexa-488 fluorescence with emission filter 530 ± 40 nm) are shown for nuclei from each of three root zones. The gates (*red rectangles*) corresponding to early (*E*), middle (*M*), and late (*L*) sub-populations of S phase are indicated for mitotic (0–1 mm) and endocycling (1–3 mm) S-phase nuclei. The heat map (**c**, *top of right plot*) indicates the *color code* for increasing nuclei density

We used in vivo EdU pulse-labeling combined with flow cytometry of isolated, fixed nuclei to generate populations from different portions of S phase as shown in Fig. 1 and reviewed by Bass et al. (2014). The DNA content in labeled nuclei from 0 to 1 mm segments ranged primarily from 2C to 4C, characteristic of cells undergoing a mitotic cell cycle (Fig. 1b, c left panel). In contrast, labeled nuclei from the 1 to 3 mm zone included a large fraction with DNA contents ranging from 4C to 8C (Fig. 1b, c middle panel), indicating a substantial sub-population of endocycling cells in this zone. The occurrence of endocycling in developing root cells is typical of many plant species [reviewed by Bass et al. (2014), Breuer et al. (2010), Takatsuka and Umeda 2014)]. Relatively little DNA replication activity was observed in nuclei isolated from 3 to 5 mm segments (Fig. 1b, c right panel). DNA contents beyond 8C were seldom observed. Labeled nuclei, which were detected using Alexa-488 (A-488) conjugated to EdU, were gate-sorted (rectangle areas in Fig. 1c) into populations representing early, middle, and late stages of the mitotic S phase (from 0 to 1 mm segments) or the endocycle S phase (from 1 to 3 mm segments). These data established the maize seedling root tip system as ideal for studying DNA replication in an intact, naturally growing, multicellular organ and for comparing replication in mitotic and endocycling plant nuclei in a well-studied genetic model system.

### 3D cytology defines early, middle, and late S replication patterns in maize

**Fig. 2:**
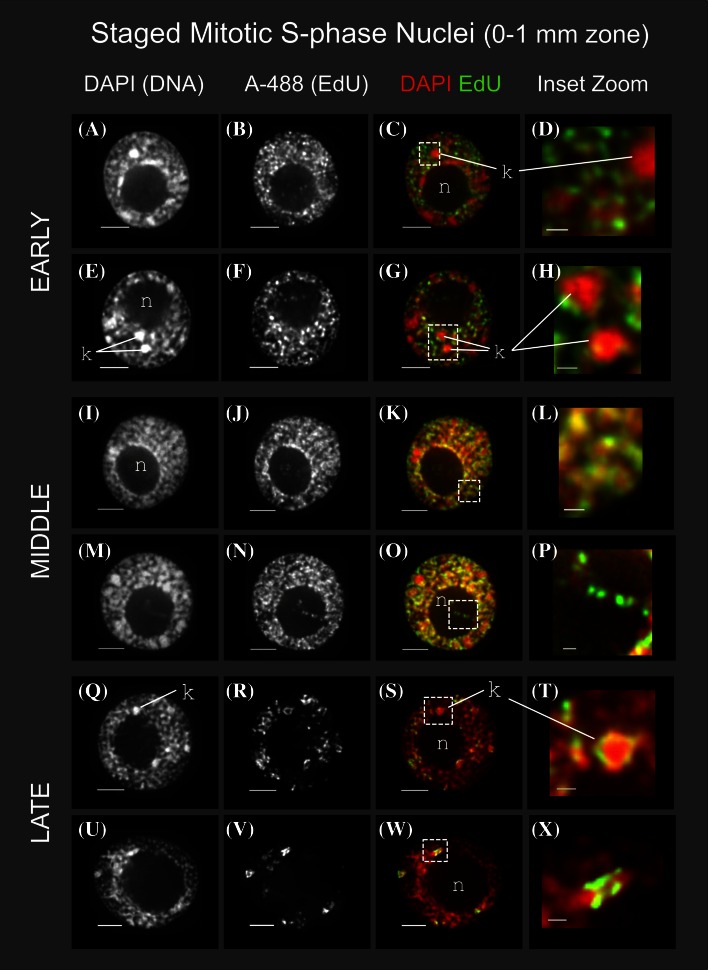
Cytology of DNA replication in staged, mitotic S-phase root tip nuclei. Nuclei were prepared as illustrated in Fig. 1 from the 0 to 1 mm section of pulse-labeled maize roots and subjected to 3D deconvolution microscopy, corrected for wavelength-dependent chromatic aberration, and displayed as *gray*-*scale* or pseudo-colored overlay as previously described (Bass et al. 2014; Howe et al. 2013). *Each row* corresponds to images from a single nucleus showing an intensity-averaged projection spanning 1 µ (3–5 Z sections, depending on Z-step distances in the original dataset). The newly synthesized DNA was imaged as A-488 fluorescence in the FITC channel, while total DNA was imaged in the DAPI channel. Two representative examples are shown for each of three sequential substages of S phase; EARLY (**a**–**h**), MIDDLE (**i**–**p**), and LATE (**q**–**x**). The location of knobs (*k*) and nucleoli (*n*) are indicated. Zoomed sections illustrate replication around, but not within, knobs in early S (**c**/**d**, **g**/**h**), overlapping signals of DAPI and A-488 in middle S bulk chromatin (**k**/**l**), detection of A-488 within the interior of the nucleolus (**o**/**p**), and bright patchy heterochromatin labeling by A-488 in late S (**s**/**t**, **w**/**x**). All *scale bars* represent 5 µ

Nuclei from the mitotic zone (0–1 mm) of maize roots were sorted into early, middle and late S phase, and subjected to 3D multiple wavelength iterative deconvolution microscopy and image analysis. This imaging technology yields high information content over a wide dynamic range using low-dose illumination to minimize sample damage and fluorescent dye bleaching [reviewed by Howe et al. (2013) and references therein]. Images of representative nuclei are shown in Fig. 2 as 1-μm projections (3–5 optical sections) from the middle of each nucleus, revealing representative DNA replication patterns during S phase in the mitotic cell cycle. Two examples of each S phase stage are shown and color overlay displays use red for DAPI and green for A-488 for visual contrast.

In early S phase, labeling occurs in numerous punctate foci distributed throughout the nucleoplasm and is mostly absent from the nucleolus (“n” in Fig. 2). The constitutive heterochromatic knobs, brightly stained with DAPI (“k” in Fig. 2), largely lack A-488 fluorescence, indicating that they are not being replicated at this time. In some nuclei, weaker, but discrete, punctate A-488 signals occur inside the nucleolus (highlighted in the dashed box in Fig. 2o, but also evident in Fig. 2b, f, j). These signals may reflect rDNA replication, suggesting that some rDNA repeats replicate inside the nucleolus or move into the nucleolus after replication during early S phase. Discontinuous A-488 staining within the nucleolus may represent asynchronous labeling of sequences within individual rDNA repeating units.

The middle S replication patterns were similar to the early S phase patterns and differed markedly from mammalian middle S patterns, which are characterized by a conspicuous transition to perinuclear and perinucleolar labeling with a concomitant reduction in distributed nucleoplasmic staining [“pattern Type III” as summarized by Zink (2006)]. Interestingly, the largest difference between maize early and middle S patterns is the relationship between the signals for EdU labeling of replicated DNA versus those for DAPI staining of total chromatin. Deconvolution images of DAPI-stained bulk interphase chromatin (excluding knobs and nucleoli) often show a patchy or wormy fiber-like pattern throughout the nucleoplasm. In early S phase, when displaying the DAPI as red and the EdU as green, we typically saw a “red + green” pattern, indicating that most of the EdU label is not coincident with bulk chromatin. In contrast, middle S phase nuclei typically exhibited a “yellow” pattern in overlay images, indicating that much of the EdU label colocalizes with bulk chromatin (cf. the early S nuclei in Fig. 2c, g to the mid S nuclei in Fig. 2k, o).

We took advantage of the wide dynamic range afforded by 3D deconvolution microscopy (linear over at least 4000 photon counts/pixel) to mathematically quantify these visually apparent differences between early and middle S phase. Specifically, we calculated the Pearson’s correlation coefficients for DAPI versus A-488 signal intensity for the entire population of voxels (volumetric pixels with X, Y, and Z dimensions) in whole nuclei image data sets as summarized for different stages of S phase in Table 1. The average Pearson’s correlation *r* value for DAPI versus A-488 signals increased from 0.77 (±0.045) for early S to 0.88 (±0.035) for middle S (Table 1), indicating that the voxel-by-voxel signal correlations per nucleus are reproducibly and significantly lower in early S phase than in middle S phase. Hence, quantitative analysis of the 3D signal distribution confirmed the visual impression that the types of chromatin being replicated in early versus middle S phase differ in their overall compaction or density as revealed by DAPI staining. This change in correlation between DAPI staining and replicative labeling as nuclei progress from early to middle S phase was unexpected, representing a key finding for which no counterpart has been documented in other plant or animal DNA replication studies.

**Table 1:** Spatial and quantitative measurements from 3D images of S-phase nuclei from roots

Root zone^a^	Stage^b^	*n*	Buffer	Volume (µm^3^)	DAPI versus FITC (Alexa-488)	
					Pearson’s correlation coefficient (*r*)^c^	COM^d^ offset (µm)
0–1	E	44	PBS	448 (±44)	0.77 (±0.045)	0.27 (±0.075)
	M	61		605 (±61)	0.88 (±0.035)	0.21 (±0.010)
	L	36		637 (±83)		
1–3	En-E	45		742 (±58)	0.78 (±0.035)	0.50 (±0.027)
	En-M	44		826 (±72)	0.85 (±0.040)	0.46 (±0.025)
	En-L	52		861 (±36)		
0–1	E	21	MBA	433 (±51)	0.75 (±0.003)	0.37 (±0.100)
	M	18		589 (±52)	0.83 (±0.064)	0.26 (±0.070)
	L	17		629 (±40)		

*MBA* meiocyte buffer A (Bass et al. 2014), *PBS* phosphate buffered saline

^a^Region of root. 0–1, 0–1 mm from end; 1–3, 1–3 mm from end

^b^Substage of S phase; E (early), M (middle), L (late), en—(endocycling)

^c^r values for Pearson’s coefficient of correlation of DAPI and FITC channel signals were determined using the softWoRx program from applied precision

^d^The calculated center of mass (COM) was determined for 3D data sets using the softWoRx program from applied precision (see also methods)

**Fig. 3:**
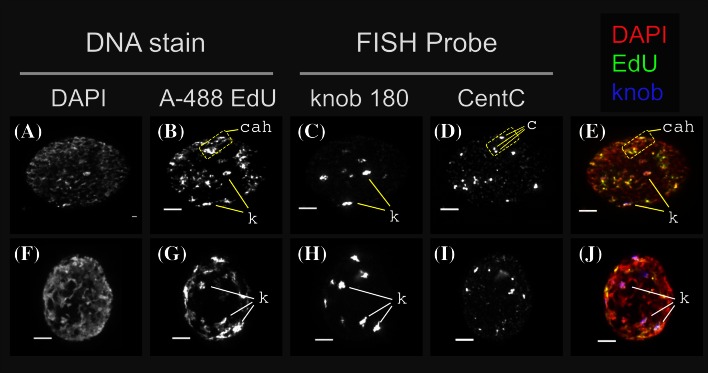
Colocalization in late S-phase nuclei of EdU signals or DNA synthesis and FISH signals for knob and centromere sequences. Flow-sorted nuclei stained with DAPI and A-488 was prepared as shown in Fig. 1 and subjected to 3D acrylamide FISH as previously described (Bass et al. 2014). Images from two different nuclei in late S phase are shown as whole-nucleus, through-focus projections showing total DNA in the DAPI channel (**a**, **f**), EdU incorporation (A-488) in the FITC channel (**b**, **g**), knob FISH probe signals (knob 180) in the rhodamine channel (**c**, **h**), and centromere-associated FISH probe signals (CentC) in the Cy-5 channel (**d**, **i**). *Pseudocolored overlay* shows the DAPI (*red*), EdU (*green*) and knobs (*blue*). The location of several individual knobs (*k*), CentC clusters (*c*), and centromere-associated heterochromatic regions (*cah*) are indicated. Oligonucleotide FISH probe (NUBI-R for knob, MCCY for centC) information is in Bass et al. (2014). All *scale bars* represent 5 µ

The nucleoplasm of late S phase nuclei (Fig. 2q–x) contained several brightly labeled, punctate foci, and the interiors of their nucleoli were not labeled. Maize knob DNA has long been known to be late-replicating and may be among the last DNA sequences to replicate during S phase (Pryor et al. 1980). We used FISH to confirm that the late-replicating DNA localizes to knob and centromere-proximal chromatin (Fig. 3). Direct-labeled oligonucleotide FISH probes specific to the 180-bp knob repeat or the 160-bp centromere-localized CentC repeat (Ananiev et al. 1998) were hybridized to late S phase nuclei prepared for 3D acrylamide FISH analysis. Whole nuclei projections from two representative images show total DNA (DAPI), DNA synthesis sites in late S phase (A-488), knob repeat sequences (knob 180) and centromere-associated foci (CentC). The maize B73 cultivar in this study has two large knobs, one each on the long arms of chromosome 5 and 7, two small knobs, and several very small knob loci that can only be reliably detected by FISH. The knobs, most of which are already evident in the DAPI images, showed the expected result of overlapping EdU labeling in late S phase.

**Fig. 4:**
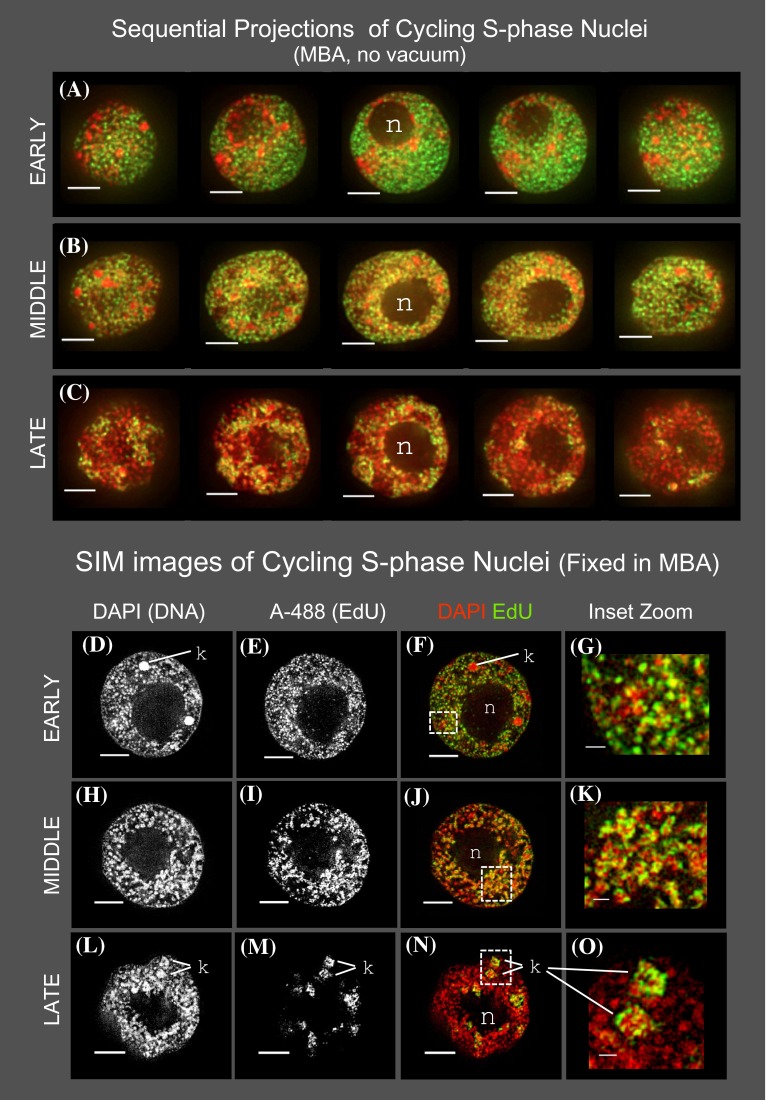
Cytology of DNA replication in staged, mitotic S-phase root tip nuclei fixed in a chromatin-preserving buffer without vacuum. Sequential projections were produced as described (Bass et al. 2014) and are shown as *pseudo-colored overlay* images. One representative nucleus is shown for each stage: **a** EARLY, **b** MIDDLE, or **c** LATE. The location of the nucleolus (*n*) is indicated. Comparison of these images to those from PBS/vacuum-fixed root tissues (Fig. 2) confirms that the early S “*red* + *green*” and middle S “*yellow*” patterns are not artifacts of vacuum or buffer. **d**–**o** Samples of nuclei fixed in MBA without vacuum were also imaged by super-resolution SIM microscopy (OMX, GE Healthcare). *Rows* and *columns* are displayed as described for Fig. 2. At this higher optical resolution, the non-overlapping early S (**d** vs. **e**–**g**) and overlapping middle S signals (**h** vs. **i**–**k**) were more detailed than but similar to those from the deconvolution images. The locations of the nucleolus (*n*) and knob (*k*) heterochromatin are indicated. All *scale bars* represent 5 µ

The high-resolution, 3D spatiotemporal patterns of DNA replication for the large maize genome showed unique as well as common patterns relative to similarly sized animal genomes. Whereas the early and late S nuclei displayed typical patterns of DNA replication, the maize middle S nuclei revealed unexpected properties. We performed control experiments (Fig. 4) to address the possibility that the PBS buffer or vacuum fixation might contribute to the unique replication patterns observed in maize nuclei. Root tips were fixed in a buffer known to preserve plant chromatin for light microscopy (buffer “MBA”, Bass et al. 2014) and in the absence of vacuum. Flow-sorted nuclei from these control samples were imaged by 3D deconvolution (Fig. 4a–c) or structured illumination super-resolution microscopy (Fig. 4d–o). In all cases, we detected the same 3D spatial DNA replication patterns (Table 1; cf. Figs. 2, 4). Hence, the findings reported were robustly detected under different preparative and imaging schema. QuickTime movies (see online supplemental materials) showing all the optical sections of representative early, middle, and late S phase nuclei (Fig. 4a–c) are provided in supplemental Table 1.

### Spatiotemporal patterns persist in endocycling nuclei

**Fig. 5:**
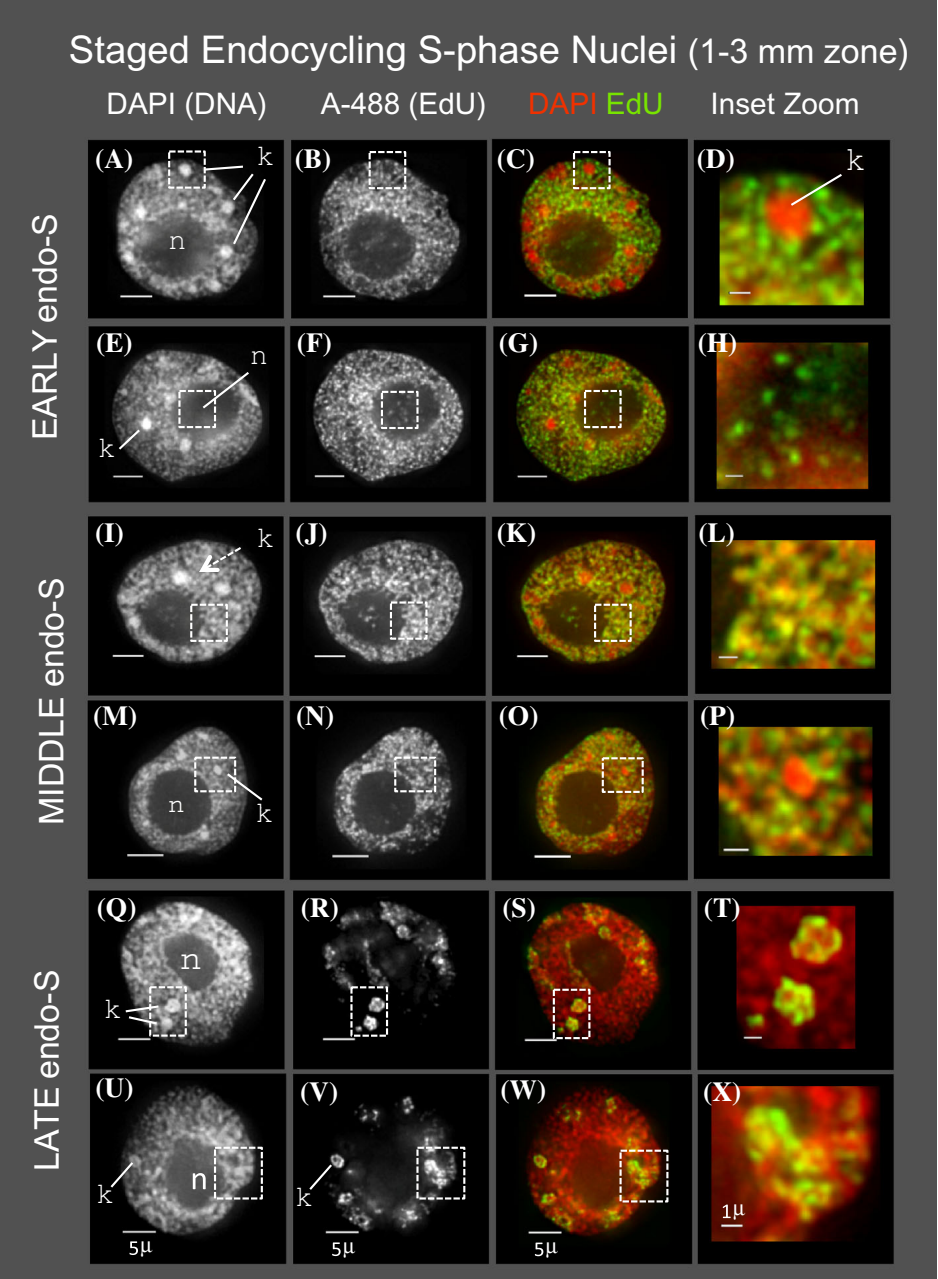
Cytology of DNA replication in endocycling root tip nuclei. Nuclei were prepared as illustrated in Fig. 1 from the 1 to 3 mm section of pulse-labeled roots and subjected to 3D deconvolution microscopy and image display as described for Fig. 2. Two representative examples are shown for each of three sequential sub-stages of endocycling S phase; EARLY endo-S (**a**–**h**), MIDDLE endo-S (**i**–**p**), and LATE endo-S (**q**–**x**). The location of knobs (*k*) and nucleoli (*n*) are indicated. Zoomed sections illustrate replication around, but not within, knobs in early endo-S (**c**/**d**) and middle endo-S (**o**/**p**), overlapping signals of DAPI and A-488 in middle endo-S bulk chromatin (**k**/**l**), detection of A-488 within the interior of the nucleolus at early endo-S (**g**/**h**), and bright patchy heterochromatin labeling by A-488 at late endo S (**s**/**t**, **w**/**x**). All *scale bars* represent 5 µ

Among the developmental fates of cells in the maize root tip is endoreduplication, as seen by flow cytometric analysis profiling (Fig. 1c; middle panel). We used the developing root tip system to compare the cytological S phase patterns of mitotic versus endocycling cells. Nuclei from the endocycling population were subjected to 3D deconvolution microscopy and image analysis as described above, with representative nuclei shown in Fig. 5. As expected, the volumes of endocycling nuclei were greater than mitotic nuclei and continued to increase (Table 1) during progression through the endocycle S phase. Observed here but not previously reported, the overall spatiotemporal pattern of DNA replication in nuclei from endocycling cells was remarkably similar at the cytological level to that for cells in the mitotic cycle, including the “red + green” pattern of early endo S (Fig. 5c, g) and the “yellow” pattern of middle endo S (Fig. 5k, o).

Replication of knobs was also similar for nuclei in the endocycle and the mitotic cycle. Specifically, knob replication was limited to late endo S phase (Fig. 5s, w) and absent from early or middle endo S phase (Fig. 5d, p). Intra-nucleolar labeling patterns were also similar, with endocycling nuclei showing faint, punctate EdU staining in the interior of the nucleolus in early and middle S phase but not in late S phase (Fig. 5, cf. nucleoli signals from EdU or color overlay panels). Finally, the Pearson’s correlation coefficient used to quantify the correlation between DAPI and A-488 showed a marked and significant increase from *r* = 0.78 (±0.035) in early endo S phase to *r* = 0.85 (±0.040) in middle endo S phase (Table 1). Together, these observations reveal a high degree of conservation of the spatiotemporal DNA replication program for nuclei in mitotic cell cycles and endocycles.

## Discussion

In this study, we characterized the spatiotemporal patterns of DNA replication using 3D cytological analysis of images collected from pulse-labeled nuclei of naturally developing maize root tips. Cytological data are inherently descriptive, but that fact does not diminish their capacity to contribute profoundly important insights into biological phenomena. Examples include the cytological proof of genetic crossing over in maize (Creighton and McClintock 1931), the semiconservative nature of DNA replication in pea (Taylor et al. 1957) and the concept of a cell cycle under genetic control [reviewed by Bryant (2014)]. Here, advanced labeling and imaging technologies were used to gain new insight into the interesting but understudied area of plant genome replication.

The early S phase staining pattern for maize nuclei resembles those described previously in other multicellular eukaryotes including pea, onion, and cell lines from several mammalian species (Nakayasu and Berezney 1989; van Dierendonck et al. 1989; O’Keefe et al. 1992; Sparvoli et al. 1994; Samaniego et al. 2002). However, two aspects of the replication patterns observed in maize differed strikingly from those reported in earlier studies. First, we did not detect conspicuous concentrations of replication activity at the nuclear or nucleolar periphery that have been repeatedly described for mouse and human cells in middle S phase [reviewed by Zink (2006)]. To confirm that peripheral labeling patterns were not discarded inadvertently during flow sorting, we examined unsorted preparations of EdU pulse-labeled nuclei and found no indication of any peripheral patterns in maize nuclei. Second, we observed and quantified distinct patterns of replication during early and middle S phase, with early replication occurring primarily in regions with weak DAPI staining while middle replication signals correlating closely with areas of strong DAPI staining.

**Fig. 6:**
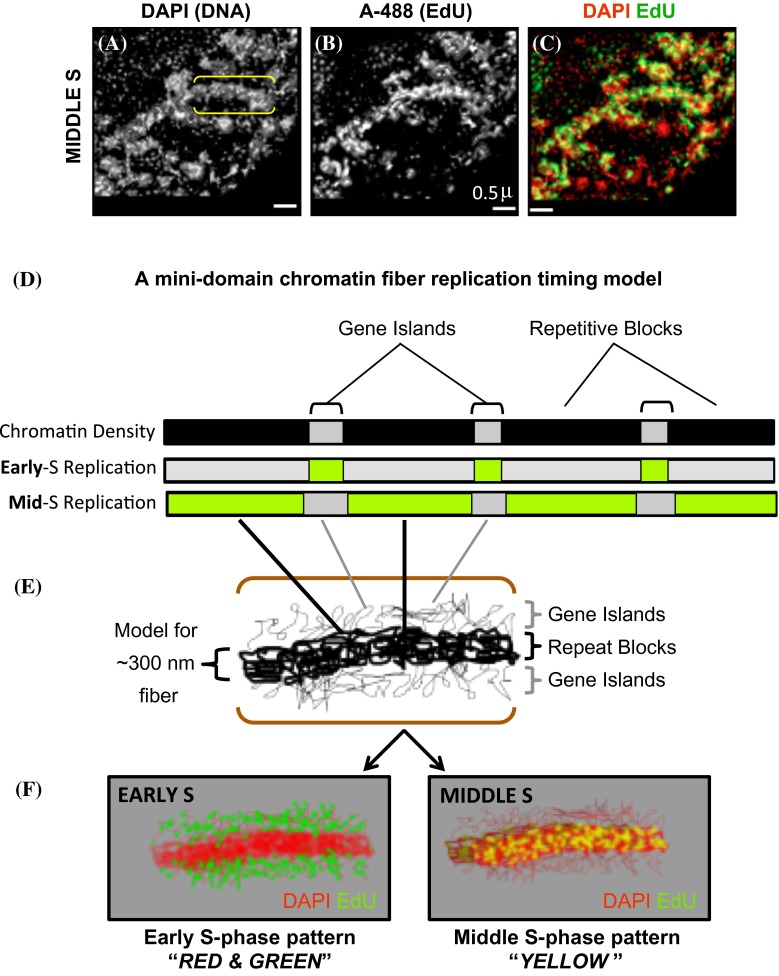
A mini-domain model for the maize DNA replication patterns during early versus middle S phase. **a**–**c** A small region from a SIM image of a nucleus in mitotic middle S phase is shown along the *top* after imaging the DAPI and EdU as described in Fig. 2. *Yellow brackets* indicate a “thick fiber” of approximately 300-nm width that can be seen in both wavelengths. **d** Schematic of genic regions (*light gray*) with alternating and larger repetitive blocks (*black*) are proposed to replicate during early (*green* sections in “Early S”) or middle (*green* sections in “Mid S”) S phase. **e** Hypothetical folding model in which the repetitive intergenic regions form the thicker most visible core of the 300-nm fiber, surrounded by less densely packaged chromatin (thin fibers *above* and *below* the 300-nm fiber) from the genic regions. **f** Diagram illustrating how the model in **e** would appear in our cytological assays of early (EARLY S) compared to middle (MIDDLE S) S phase. The organization depicted in **e** could appear as the “*RED* and *GREEN*” pattern of bulk chromatin seen in Early S (e.g. Figs. 2c, g, 4a, 5c, g), or the “*YELLOW*” pattern of bulk chromatin seen in Middle S (e.g. Figs. 2k, o, 4b, 5k, o)

To gain insight into why the incorporated label does not coincide with DAPI staining during early S phase but does during middle S phase, we considered the structure of the maize genome. Maize genes mostly exist singly or in small clusters, referred to as “gene islands,” separated by blocks of intergenic sequence ranging in size from several to several 100 kbp and composed primarily of different families of retroelements that are distinct from classical heterochromatic repeats (Liu et al. 2007; SanMiguel et al. 1996; Schnable et al. 2009). Given this basic organizational pattern, we propose a “mini-domain model” as summarized in Fig. 6 to relate replication timing to genome structure in the euchromatic arms of a typical maize chromosome. Images of DAPI-stained maize interphase chromatin often reveal a recurring pattern of thick fibers about 300 nm wide. In our model, these thick fibers (Fig. 6a, bracketed region) would represent locally compacted repetitive blocks of DNA (Fig. 6d, e). Combining the genome organizational pattern with the common tendency for open, transcriptionally active chromatin to replicate early, we hypothesize that open, genic chromatin exists as low density projections from the 300-nm fibers, and that this low density chromatin is preferentially replicated in early S phase (Fig. 6; thin fibers in 6e, green dots in 6f Early S panel). The low density chromatin would be barely visible in DAPI images, but prominently labeled by EdU. In contrast, we propose that repeat blocks constitute most of the chromatin in 300-nm fibers, which are more brightly stained by DAPI and replicate mainly in middle S phase. The two types of euchromatin, genic versus intergenic, would be in close proximity to each other, but with limited spatial overlap, thereby producing the “red and green” pattern characteristic of early S phase and the “yellow” pattern characteristic of middle S phase.

An alternative model in which different gene activity states, active verses inactive, result in different replication timing, early verses middle, respectively, could also explain our findings. Such a model is not mutually exclusive with the mini-domain model, as one could envision gene islands with one or more active genes conditioning early replication, whereas chromatin in transcriptionally inactive regions, such as silent genes or blocks of repeats, are more compacted and condition middle S replication. In either case, our data and model are largely congruent with the developing paradigm of the coexistence of multiple chromatin states (Roudier et al. 2011; Julienne et al. 2013; Pope and Gilbert 2013) rather than the more traditional view in which chromatin exists in either an open active state or a closed inactive state.

One of the implications of our model is that it may provide an explanation for the apparent absence of a distinct set of sequences replicating during middle S phase in Arabidopsis (Lee et al. 2010), a species with much less repetitive DNA than maize. This hypothesis could be tested using 3C mapping methods to examine the coupling of replication timing domains with chromatin interaction data, as recently reported in mammalian systems (Pope and Gilbert 2013).

Given that endocycling nuclei enter a second S phase without the intervening mitosis of a typical cell cycle, there is no a priori reason to presume that mitotic and endocycling S phases would have similar replication programs. However, we found a remarkable congruence between S phase in the mitotic cycle and the endocycle for all of the examined cytological features, including the progressive increase in nuclear volume (Table 1). Thus, the determinants of these cytological patterns must be independent of, or able to by-pass, mitotic processes such as chromatin condensation, sister-chromatid separation, and nuclear-envelope dissolution and reformation. In yeast and mammals, replication timing for a particular sequence, region or domain can sometimes vary, reflecting changes in chromatin state, epigenomic features or transcriptional activity [reviewed by Aparicio (2013)]. Nevertheless, we showed that the cytological events associated with mitotic replication and the endocycle are visibly and quantitatively very similar. We cannot exclude the possibility that certain sequences replicate at different times, or undergo differential replication, in mitotic versus endocycling nuclei. Most events of this type would be beyond the detection limits of cytological analyses and would be revealed only by detailed molecular analysis of replication timing profiles. However, our data on nuclear volume (Table 1) and DNA content estimates from flow cytometry are consistent with a single, complete replication of the entire maize genome during endo S phase.

In summary, by careful measurement of the global relationship between DNA synthesis and bulk DNA density in 3D quantitative deconvolution image reconstructions, we defined aspects of DNA replication in maize not previously described in other eukaryotes. Several features of DNA replication were apparent at the whole nucleus scale. Maize DNA replication activity is widely distributed in the nucleoplasm during both early and middle S phase, and does not show the perinuclear and perinucleolar patterns characteristic of mammalian middle S phase. Statistical analysis of the 3D data showed that maize DNA synthesized during middle S phase tightly colocalizes with the brightly stained DAPI signals in the nucleoplasm, a pattern sharply distinct from the early S phase pattern of labeling in decondensed regions with only weak DAPI signals. This partitioning may reflect an interspersed pattern of chromatin states that is particularly well resolved in maize. In addition, we found that endocycling nuclei exhibit the same cytological progression within S phase as nuclei in the mitotic cell cycle. A final and highly significant aspect of this study derives from the use of nuclei isolated from cells pulse-labeled and fixed while in a naturally developing organ of the plant. This system does not rely on cell lines, artificial growth conditions or inhibitory chemicals. As such, it provides an important benchmark for translating knowledge of DNA replication between cultured cells and the whole organism, while establishing a foundation for future genetic, epigenetic and genomic analyses of plant DNA replication.

## Experimental procedures

### Plant material

*Zea mays* B-73 seeds were imbibed in running water overnight, and germinated in sterile Magenta boxes (Sigma-Aldrich) containing a damp paper towel under constant light at 28 °C. After 3 days, the seedlings were immersed in sterile water containing 25 μM 5-ethynyl-2′-deoxyuridine (EdU, Life Technologies) for 20 min with gentle agitation. After washing with sterile water, the terminal 0–1 and 1–3 mm root segments were excised from primary and seminal roots. The terminal and subapical segments were separately fixed in 1 % formaldehyde in 1× phosphate-buffered saline (PBS) for 15 min with the first 5 min under vacuum, then washed in PBS three times, and finally snap frozen in liquid nitrogen. As a control, a few batches of cut root segments were alternatively fixed in 1 % formaldehyde in Meiocyte Buffer A [MBA from Bass et al. (2014)] for 2 h without vacuum.

### Nuclei isolation

The frozen roots were ground in a cell lysis buffer (CLB: 15 mM Tris–HCl pH 7.5, 2 mM EDTA, 80 mM KCl, 20 mM NaCl, 0.1 % Triton X-100, 15 mM β-mercaptoethanol, pH 7.5) in a small commercial food processor at 4 °C. The ground cell suspension was incubated, filtered, and centrifuged as previously described (Lee et al. 2010). Isolated nuclei were washed in CLB-wash buffer (CLB without EDTA and without β- mercaptoethanol) and centrifuged at 200×*g* for 5 min at 4 °C. The incorporated EdU was visualized after conjugation with A-488 using a Click-iT EdU Alexa fluor 488 kit (Life Technologies). The nuclei were incubated in the Click-iT reaction cocktail for 30 min according to the manufacturer’s instructions, washed with two volumes of CLB, and pelleted. Finally, the nuclei were resuspended in CLB containing 2 μg/mL DAPI, and filtered through a 20-μm nylon filter (Partec) before flow cytometry and sorting.

### Flow cytometry and sorting

Isolated nuclei were sorted and recovered with an InFlux flow cytometer (BD Biosciences) as previously described (Lee et al. 2010; Bass et al. 2014), except the nuclei were sorted into 2× CLB without β-mercaptoethanol. Nuclei prepared from the 0–1 mm root segments were sorted using sub-stage gates to collect populations of EdU/A-488-labeled nuclei with DNA contents in a defined windows corresponding to early, middle and late S-phase between 2C and 4C populations (Fig. 1). Endocycle nuclei were sorted by similar procedures, except that 1–3 mm root segments were used and collections were made in windows between the 4C and 8C populations. Flow cytometry data was analyzed using FlowJo software (Tree Star Inc., Ashland, OR, USA).

### Preparation of samples for microscopy

Flow-sorted nuclei were obtained as described above, gently pelleted and stored in 1× PBS buffer at 4 °C and imaged within weeks or months of fixation with no apparent degradation in morphology or signal. Prior to 3D deconvolution microscopy imaging, fixed nuclei were re-stained with 5 μg/mL DAPI for 10 min, briefly pelleted, washed with 1× PBS supplemented with 1 mM DTT, placed on glass slides, mounted in VectaShield (H-1000, Vector laboratories), and sealed under 1.5 coverslips for 3D deconvolution microscopy imaging. Three-dimensional FISH was carried out using the 3D acrylamide FISH method with fluorescent oligonucleotide probes as described by Howe et al. (2013, and references therein).

### Three-dimensional image data collection, processing, and display

Images were recorded with a DeltaVision 3D deconvolution microscope (Applied Precision) with an Olympus IX-70 wide-field microscope and a 60× NA 1.4 PlanApo (Olympus) oil-immersion lens with 1.5X magnification. Image data were oversampled in the *X*, *Y* and *Z* dimensions with typical *XYZ* voxel dimensions of 0.07 × 0.07 × 0.2 μm. Grayscale images were recorded on a cooled-CCD camera and 3D datasets were subjected to three-dimensional iterative deconvolution (Chen et al. 1995), and chromatic aberration correction prior to analysis and measurements. The resulting 3D data sets were then cropped around individual whole nuclei prior to 3-D modeling and spatial analysis. The images presented were adjusted for brightness and contrast by linear scaling, and multiple-wavelength images were pseudo-colored. Through-focus projections were made using the ‘average intensity projection’ mode. Sequential projections were made by dividing the total number of optical sections per nucleus by five and making through-focus average projections for each fifth of the nucleus. Pseudo-colored overlays were adjusted for brightness and contrast using linear scaling with red for DAPI and green for A-488. High resolution structured illumination OMX microscopy (courtesy of A. Quintanilla, Applied Precision Inc.,) was carried out using slides prepared by our group.

### Measurements of 3-D image data

The program EditPolygon was used to trace the edges of the nucleus (DAPI image) manually, drawing circles on each optical section for a given nucleus. The VolumeBuilder program was used to connect the polygon series into a 3-D object with a closed and continuous surface. The 3-D object files were used to measure the nuclear volumes and center of intensity (intensity weighted center of space) for the objects in the DAPI and A-488 images. The distance between the centers of intensity for DAPI and A-488 datasets was calculated using standard Euclidian distance measurements. Measurements reported in Table 1 were tabulated separately for each combination of fixation/buffer conditions (PBS vs. MBA) or for each type of microscopy (3D deconvolution/DV vs. structured illumination/OMX).

## Electronic supplementary material

Supplementary Table 1 3D movie files of optical sections from image datasets of DAPI and A488 at Early-, Middle-, or Late-S phase maize root tip nuclei (PDF 172 kb)

Supplementary material 2 ZmRootBFA_EarlyS__DAPI (MOV 623 kb)

Supplementary material 3 ZmRootBFA_EarlyS__FITC (MOV 1165 kb)

Supplementary material 4 ZmRootBFA_EarlyS_DFmg (MOV 1329 kb)

Supplementary material 5 ZmRootBFA_EarlyS_DFrg (MOV 1234 kb)

Supplementary material 6 ZmRootBFA_EarlyS_DF.dv (DV 5384 kb)

Supplementary material 7 ZmRootBFA_MidS__DAPI (MOV 879 kb)

Supplementary material 8 ZmRootBFA_MidS__FITC (MOV 894 kb)

Supplementary material 9 ZmRootBFA_MidS_DFrg (MOV 1043 kb)

Supplementary material 10 ZmRootBFA_MidS_DFmg (MOV 1088 kb)

Supplementary material 11 ZmRootBFA_MidS_DF.dv (DV 4148 kb)

Supplementary material 12 ZmRootBFA_LateS__DAPI (MOV 1095 kb)

Supplementary material 13 ZmRootBFA_LateS__FITC (MOV 998 kb)

Supplementary material 14 ZmRootBFA_LateS_DFrg (MOV 1233 kb)

Supplementary material 15 ZmRootBFA_LateS_DFmg (MOV 1335 kb)

Supplementary material 16 ZmRootBFA_LateS_DF.dv (DV 6227 kb)

## Abbreviations

EdU: 5-Ethynyl-2′-deoxyuridine
AF-488 or A-488: ALEXA-fluor 488
DAPI: 4′,6-Diamidino-2-phenylindole dihydrochloride
FITC: Fluorescein isothiocyanate
