# Supplementary material for: Defining multiple, distinct, and shared spatiotemporal patterns of DNA replication and endoreduplication from 3D image analysis of developing maize (Zea mays L.) root tip nuclei
Source: Plant Mol Biol. 2015 Sep 22;89(4-5):339–51. doi: 10.1007/s11103-015-0364-4 (PMC4631726; doi:10.1007/s11103-015-0364-4)
Supplement: Supplementary file 1 — 3D movie files of optical sections from image datasets of DAPI and A488 at Early-, Middle-, or Late-S phase maize root tip nuclei (PDF 172 kb) [file 11103_2015_364_MOESM1_ESM.pdf]

Supplemental Table 1. 3D movie files of optical sections from image datasets of DAPI and A488 at Early-, Middle-, or Late-S phase maize root tip nuclei.

S-phase substage	**Wavelength, filter sets**	**Fluorescent dye (molecules imaged)**	**Optical section colors**	**Movie or Source file**
EARLY (Fig 4A)	DAPI	DAPI (total DNA)	grey-scale	ZmRootBFA_EarlyS__DAPI
	FITC	Alexa-488 (EdU-labeled DNA)	grey-scale	ZmRootBFA_EarlyS__FITC
	DF, DAPI & FITC	DAPI (DNA) & Alexa-488 (EdU)	pseudo-color (mg = magenta & green; rg = red & green)	ZmRootBFA_EarlyS_DFmg<br>ZmRootBFA_EarlyS_DFrg
	DAPI & FITC	DAPI (DNA) & Alexa-488 (EdU)	DeltaVision 3D source file, (.dv)	ZmRootBFA_EarlyS_DF.dv
MIDDLE (Fig. 4B)	D, DAPI	DAPI (total DNA)	grey-scale	ZmRootBFA_MidS__DAPI
	F, FITC	Alexa-488 (EdU-labeled DNA)	grey-scale	ZmRootBFA_MidS__FITC
	DF, DAPI & FITC	DAPI (DNA) & Alexa-488 (EdU)	pseudo-color (mg = magenta & green; rg = red & green)	ZmRootBFA_MidS_DFrg<br>ZmRootBFA_MidS_DFmg
	DAPI & FITC	DAPI (DNA) & Alexa-488 (EdU)	DeltaVision 3D source file, (.dv)	ZmRootBFA_MidS_DF.dv
LATE (Fig. 4C)	D, DAPI	DAPI (total DNA)	grey-scale	ZmRootBFA_LateS__DAPI
	F, FITC	Alexa-488 (EdU-labeled DNA)	grey-scale	ZmRootBFA_LateS__FITC
	DF, DAPI & FITC	DAPI (DNA) & Alexa-488 (EdU)	pseudo-color (mg = magenta & green; rg = red & green)	ZmRootBFA_LateS_DFrg<br>ZmRootBFA_LateS_DFmg
	DAPI & FITC	DAPI (DNA) & Alexa-488 (EdU)	DeltaVision 3D source file, (.dv)	ZmRootBFA_LateS_DF.dv

Table Notes:

Three different nuclei (Fig. 4A-C), displayed in various movie formats (see online supplemental materials), were from Meiocyte BufferA (MBA) plus formaldehyde-fixed 0-1 mm section of pulse-labeled maize seedling root tips. The 3D deconvolution datasets were cropped in 3 dimensions around each nucleus. Exported quicktime movies step through the optical Z-sections the nuclei, shown as grey-scaled or pseudo-color as listed in the table. The image wavelengths are designated “D” for the DAPI channel images (DAPI dye) showing total DNA/chromatin or “F” for the FITC channel images (Alexa-488 dye) showing locations of EdU incorporation into newly synthesized DNA incorporated during the EdU labeling pulse. Colored sections use either red or magenta for the DAPI image, and green for the FITC/A-488 image. Also included are links to the source DeltaVision datasets (.dv files). Scale bars are 1 micron.
